# Demineralized Bone Matrix Add-On for Acceleration of Bone Healing in Atypical Subtrochanteric Femoral Fracture: A Consecutive Case-Control Study

**DOI:** 10.1155/2016/4061539

**Published:** 2016-02-28

**Authors:** Noratep Kulachote, Paphon Sa-ngasoongsong, Norachart Sirisreetreerux, Pongsthorn Chanplakorn, Praman Fuangfa, Chanyut Suphachatwong, Wiwat Wajanavisit

**Affiliations:** ^1^Department of Orthopedics, Faculty of Medicine Ramathibodi Hospital, Mahidol University, Bangkok, Thailand; ^2^Department of Radiology, Faculty of Medicine Ramathibodi Hospital, Mahidol University, Bangkok, Thailand

## Abstract

*Background.* Delayed union and nonunion are common complications in atypical femoral fractures (AFFs) despite having good fracture fixation. Demineralized bone matrix (DBM) is a successfully proven method for enhancing fracture healing of the long bone fracture and nonunion and should be used in AFFs. This study aimed to compare the outcome after subtrochanteric AFFs (ST-AFFs) fixation with and without DBM.* Materials and Methods*. A prospective study was conducted on 9 ST-AFFs patients using DBM (DBM group) during 2013-2014 and compared with a retrospective consecutive case series of ST-AFFs patients treated without DBM (2010–2012) (NDBM group, 9 patients). All patients were treated with the same standard guideline and followed up until fractures completely united. Postoperative outcomes were then compared.* Results*. DBM group showed a significant shorter healing time than NDBM group (28.1 ± 14.4 versus 57.9 ± 36.8 weeks, *p* = 0.04). Delayed union was found in 4 patients (44%) in DBM group compared with 7 patients (78%) in NDBM group (*p* > 0.05). No statistical difference of nonunion was demonstrated between both groups (DBM = 1 and NDBM = 2, *p* > 0.05). Neither postoperative infection nor severe local tissue reaction was found.* Conclusions*. DBM is safe and effective for accelerating the fracture healing in ST-AFFx and possibly reduces nonunion after fracture fixation. Trial registration number is TCTR20151021001.

## 1. Introduction

Atypical femoral fractures (AFFs) are one of the current challenging topics in orthopaedic trauma and mostly related to long-term bisphosphonates (BPs) use from prevention of fragility fracture [1, 2]. Although the exact etiology of AFFs still has not been discovered, epidemiologic studies showed that most of AFFs had delayed fracture healing problem resulting in a great risk of nonunion and implant failure [1, 2]. This is mainly because AFFs have an intrinsic poor healing capacity from severely suppressed bone turnover (SSBT) that is commonly associated with long-term bisphosphonates and steroids use [1, 2]. Moreover, many other factors, such as age, comorbid diseases, ongoing medications, and fracture location in subtrochanteric region, also contribute to the prolong fracture healing capacity and, therefore, poor postoperative outcome. In 2010, a task force of American Society of Bone and Mineral Research (ASBMR) recognized this problem by concluding the related studies and then gave a recommendation for AFFs treatment including medicinal management, such as stopping bisphosphonates and prescribing teriparatide if possible, and surgical treatment with intramedullary nail [1]. However, recent studies have still shown a high rate of complications following this treatment strategy, with 56.5% of patients having delayed union [3], and 46% of them requiring revision surgery [4]. Therefore, using local adjunctive therapy, such as bone graft (autograft or allograft) or orthobiologic agent, during the fracture fixation procedure to enrich the biological substance and cytokines in a fracture site, would be an appropriate strategy to solve this problem.

Demineralized bone matrix (DBM), one of the common orthobiologic agents, is an osteoconductive and osteoinductive allograft product, which has proven its safety and efficacy to enhance bone healing in both fracture and nonunion surgeries of the long bone [5–8]. Although autologous bone graft was considered a gold standard for biologic augmentation, using DBM has many advantages such as no limitation of quantity, more reliability in osteoinductive property than autologous bone graft taken from elderly patients in a condition with drug-induced SSBT, shorter operative time, and absolutely avoiding the risk of additional procedure and no donor-site morbidity [8, 9]. Therefore, we hypothesized that an augmentation with DBM in subtrochanteric atypical femoral fracture (ST-AFFs) would improve the fracture healing capacity and postoperative outcome.

## 2. Materials and Methods

### 2.1. Study Design and Inclusion and Exclusion Criteria

This study was a single-centered prospective cohort study in a medical university hospital, between 2013 and 2014, which was compared to a retrospective consecutive case series within the same center from the earlier 3-year period. The prospective arm (DBM group) directly followed the retrospective arm (NDBM group) when the study was initiated. The inclusion criteria were the patients who were (1) newly diagnosed as AFFs regarding to the criteria of ASBMR task force 2013 [2], (2) having the fracture located on the subtrochanter defined as the area of 5 centimeters below to the lesser trochanter, (3) aged over 55 years, and (4) having no history of previous allergy to DBM. The exclusion criteria were (1) other pathological fragility fractures such as fractures from metastasis or primary tumor or simple osteoporotic fractures apart from AFFs, (2) history of high-energy trauma such as motorcycle accident, and (3) fracture location distal to 5 cm below lesser trochanter. Prior approval was obtained from our Institutional Board Review (Protocol ID 07-58-45) and informed consent was obtained from all patients in the prospective arm group, who participated in the study, before the surgery was scheduled.

### 2.2. Surgical Intervention and Postoperative Protocol

After diagnosing as ST-AFFs, the patients were admitted to the orthopaedic trauma ward for preoperative medical clearance and surgical planning. The operations were all done within 72 hours after admission. Specific preoperative workup protocol for AFFs was applied including clinical assessment and relevant investigations [10]. Clinical assessment included history taking of mechanism of falling, prodromal pain, associated comorbid illnesses, duration of bisphosphonate use, and risk factors for other metabolic bone diseases such as hypothyroid, chronic steroid usage, and renal failure. Relevant investigations included laboratory workup for metabolic bone disease, bone mass density assessment, radiographs of both femurs, and bone scan or magnetic resonance imaging if incomplete fracture was suspected on the contralateral side [10]. The surgery was all performed by one of the teams of experienced orthopaedic trauma surgeons (Noratep Kulachote, Paphon Sa-ngasoongsong, and Norachart Sirisreetreerux), under general or spinal anesthesia based on anesthesiologist decision. Before the introduction of this study, our treatment guideline for displaced ST-AFFs followed the recommendations from ASBMR task force 2010 [1] which indicated stopping bisphosphonate immediately after diagnosis and stabilizing fracture with full-length intramedullary nail. The fracture was reduced by mini-open and clamp-assisted reduction technique [11] in lateral decubitus position on a radiolucent operative table [12] and fixed with a standard long cephalomedullary nail with 2 distal interlocking screws. Neither bone grafting nor local orthobiologic agent was added. After the study protocol introduction, the fracture was treated with the same surgical technique and using the same implant, but 1-2 mL of DBM (DBX®, Synthes, USA) was filled into the fracture site depending on the extent of intraoperative fracture gap. All surgical wounds were closed without any drain in order to preserve DBM and hematoma that surrounded fracture site.

Postoperative care and rehabilitation were managed by the same protocol. Postoperative pain was controlled by pain medications except nonsteroidal inflammatory drugs (NSAIDs) in order to avoid delay in bone healing [13]. Cold pack was applied on the fracture site every 4 hours for one day. Prophylactic intravenous antibiotic was administered for 24 hours. Sutures were removed two weeks postoperatively. Intermittent pneumatic pump was applied on both legs. Active ankle, knee, and hip motions were advised to prevent venous thromboembolism. The patients were allowed to have partial weight bearing as tolerated on the injured leg with gait aids a few days after the operation, followed by full weight bearing when the fracture healing was demonstrated on the follow-up radiographs. Daily 20 mcg subcutaneous injection of teriparatide was prescribed postoperatively for 6 months. If the patients refused injection or had the contraindication or precaution for teriparatide (such as drug hypersensitivity, history of skeletal malignancy or bone metastasis, metabolic bone disease other than osteoporosis, history of teriparatide use more than 2 year, hypercalcemic disorder, urolithiasis and hypercalciuria, and having possible drug interaction such as digoxin, hydrochlorothiazide, and furosemide), strontium ranelate was prescribed instead. However, if the patient had severe renal impairment, such as end-stage renal disease, no anabolic treatment was given. The radiographs were taken intervals every 4–6 weeks to check for the fracture healing progression. All patients were followed until the fracture completely united.

### 2.3. Data Collection and Outcome Measurement

Demographic data such as age, gender, weight, height, the side of injury, and comorbid disease were collected. Body mass index (BMI) was then calculated from weight and height. Concurrent medications that were associated with AFFs (such as bisphosphonates, statins, steroids, and proton pump inhibitors) and the duration of bisphosphonate exposure were also recorded. Preoperative laboratory data, such as hemoglobin (Hb), albumin, creatinine clearance, calcium, inorganic phosphate, total 25-OH vitamin D, total procollagen type 1 N-terminal propeptide (P1NP), collagen type 1 C-telopeptide (*β*-cross laps), and parathyroid hormone level (PTH), were recorded.

Postoperative radiographs were evaluated by 3 authors (Noratep Kulachote, Paphon Sa-ngasoongsong, and Praman Fuangfa) for fracture reduction alignment, neck-shaft angle of both normal and injured sides, and status of fracture union. Radiographic healing was defined as bridging callus on three out of four cortices as determined on the anteroposterior and lateral views [14]. Fracture healing after 6 months was defined as delayed union [15], while fractures without increased callus formation after 6 months on follow-up radiographs or those who had hardware failure were defined as nonunion [14].

Primary outcome was the healing time calculated from the duration between the first operative date and the time of diagnosis of fracture healing on radiographs. Secondary outcomes were the status of union, delayed union, and nonunion.

### 2.4. Statistical Analysis

Statistical analysis was performed using Statistical Package of Social Sciences (SPSS) software version 18.0. Continuous data were presented as mean and standard deviation and compared with *t*-test. Categorical data were presented as proportion and compared with Fisher's exact test or Chi-square test as appropriate. Significant difference was considered if *p* value < 0.05.

## 3. Result

A total of 18 patients (17 females and one male) were enrolled into this study (nine patients in each of NDBM and DBM group). Demographic data and clinical results were shown on Tables 1 and 2. The average age was 67 years (range 56–81 years). The mean BMI was 23.2 kg/m^2^ (range 18.2–28.7 kg/m^2^). Three patients had diabetic mellitus (2 in NDBM group and 1 in DBM group) and two patients had rheumatoid arthritis (both in DBM group). Seventeen patients (94%) had history of bisphosphonate use and the mean duration of the bisphosphonate exposure was 8.2 years (range 1.5–15 years). One patient (6%, number 8), who did not receive bisphosphonate, had long-term steroids due to rheumatoid arthritis. There was no significant difference between age, gender, the side of injury, BMI, comorbid disease, ongoing medications, duration of bisphosphonate exposure, and preoperative laboratory values between both groups (*p* > 0.05 all). However, the follow-up time was significantly longer in the NDBM group compared with the DBM group (*p* = 0.04).

**Figure 1:**
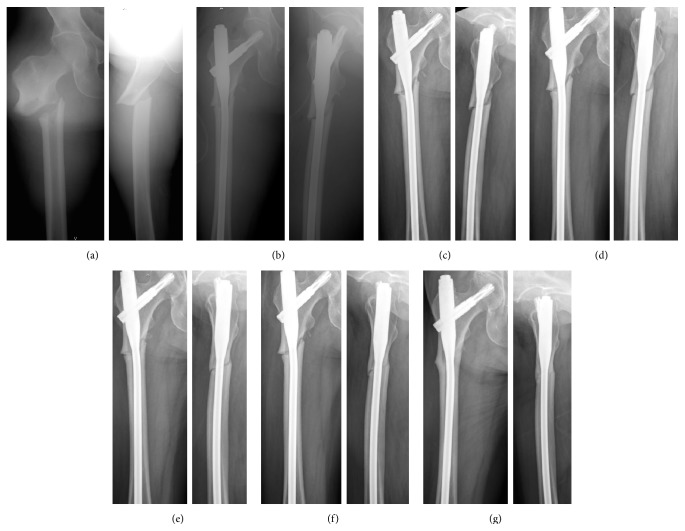
Radiographs from case examples with atypical subtrochanteric femoral fracture (ST-AFF) treated without using demineralized bone matrix (NDBM group). Preoperative (a) and immediate postoperative (b) radiographs showed ST-AFF treated with cephalomedullary nail. Follow-up radiographs after 2 months (c), 4 months (d), and 6 months (e) showed very minimal callus formation. However, the fracture still had healing progression after 8 months (f) and finally completely united after 14 months postoperatively (g).

**Figure 2:**
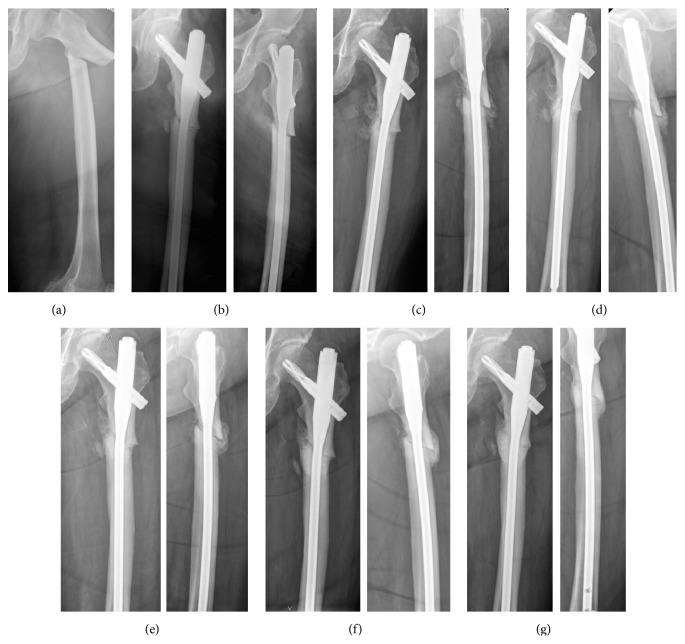
Radiographs from case examples with atypical subtrochanteric femoral fracture (ST-AFF) treated using demineralized bone matrix (DBM group). Preoperative (a) and immediate postoperative (b) radiographs showed ST-AFF treated with cephalomedullary nail. Follow-up radiographs after 3-month (c) and 4.5-month (d) period demonstrated the appropriate callus formation on anterior, posterior, and medial cortex and therefore was considered healed fracture. The fracture remodeling without complication was confirmed on 6-month, 8-month, and 11-month radiographs postoperatively.

Postoperative outcomes were shown in Table 3. There was no significant difference in postoperative fracture reduction alignment and neck-shaft angle between both groups (*p* > 0.05 all). Postoperative teriparatide injection was given in 5 patients in non-DBM group and 6 patients in DBM group. Strontium ranelate was given in 3 patients in non-DBM group and 2 patients in DBM group. One patient in each group did not receive anabolic agent postoperatively. There was no significant difference between the distribution of postoperative medication in both groups (*p* = 1.00). The DBM group showed a significant shorter healing time compared with the NDBM group (28.1 ± 14.4 weeks versus 57.9 ± 36.8 weeks, *p* = 0.04). Subgroup analysis showed that the DBM group with and without postoperative teriparatide had nonsignificantly shorter healing time compared to the NDBM group (*p* = 0.09 and 0.13, resp.). Case examples from NDBM group (case number 4 from Table 2) and from DBM group (case number 16 from Table 2) were shown in Figures 1 and 2, respectively. Delayed union occurred in 4 patients (44%) in the DBM group and 7 patients (78%) in the NDBM group (*p* = 0.33). Atrophic or oligotrophic nonunion was developed in 2 patients (22%) in the NDBM group and one patient (11%) in the DBM group (*p* = 1.00). All patients with nonunion were successfully treated with nail removal and open reduction and internal fixation with either 95-degree angle blade plate or proximal femur locking compression plate and augmentation with autologous bone graft and DBM. Neither surgical site infection nor severe local tissue reaction was found in this study.

## 4. Discussion

Management of atypical femoral fractures (AFFs) is a challenging task in orthopaedic trauma, mainly due to poor fracture healing property related to severely suppressed bone turnover [1, 2]. Additionally, many contributing factors, such as age, comorbid illnesses, concurrent medications, and fracture location in subtrochanter area, may negatively affect the fracture healing and result in delayed union, nonunion, and implant failure. Moreover, the AFFs patients are commonly associated with long-term bisphosphonates or steroids use that directly prolonged the fracture healing and remodeling process, especially when direct bone healing process was expected (such as the plate and screw fixation). Therefore, ASBMR task force had recommended the specific treatment guideline for AFFs including appropriate medical management and surgical fixation with an intramedullary nail [1]. However, recent studies have demonstrated that this strategy still resulted in a high rate of delay union and reoperation due to fracture healing complications [3, 4, 15, 16]. Therefore, using local adjunctive therapy with orthobiologic agent, during the fracture fixation surgery to enrich the biological substances and cytokines in fracture site, should be a key to success to solve this problem. Unfortunately, the autologous bone graft, a gold standard biologic agent, would not be an appropriate choice in AFFs patients due to the limitation of quantity, unreliable osteoinductive property from prolonged drug-induced osteoclastic suppression, need for additional operative procedure, and risk of donor-site morbidity [8, 9]. On the other hand, demineralized bone matrix (DBM), a common orthobiologic agent, which had demonstrated its safety and efficacy in treatment of both fracture and nonunion of the long bone surgeries [5–8], should be useful in enhancing bone healing in AFFs. Therefore, this study aimed to compare the outcome of subtrochanteric atypical femoral fracture (ST-AFFs) treated with a standard guideline with and without using demineralized bone matrix (DBM), as an add-on orthobiologic agent, in terms of the healing time and fracture healing complication.

The result from this study, though, showed an incidence of delayed union after treatment of ST-AFFs by a standard guideline as 78% (7 patients in NDBM group), which was higher than previous studies (43–57%) [3, 4, 15, 16]. This may be due to the fact that our study population focused only on subtrochanteric fractures which typically have poorer healing capacity than diaphyseal fractures due to a relative lack of blood supply and higher stress load on the fracture site, resulting in a high incidence of delayed union in this series. However, the most important finding in this study was that we could demonstrated a significant improvement in fracture healing time in the DBM group compared to the NDBM group (*p* = 0.04), which implied that the add-on DBM resulted in a significantly increased fracture healing capacity by improving osteogenic and osteoinductive properties by enriching the biological substances and cytokines and, therefore, promoting the bone generation in AFFs after treatment with intramedullary nailing. However, we could not demonstrate the significant improvement on the incidence of delayed union (44% in DBM group versus 77% in NDBM group, *p* = 0.33) and nonunion (11% in DBM group versus 22% in NDBM group, *p* = 1.00). This could be explained by the effect of small sample size and confounding factors which affected the fracture healing such as age, comorbid illness, and postoperative medications [15, 17].

Our study also had several limitations. First, we had a small number of patients due to the uncommon presentation of ST-AFFs. Second, there were no comparative data of the non-AFF treatment that needs teriparatide or strontium supplement because the fracture healing potential in those patients was still good, and we also did not use any anabolic medications to enhance bone healing in our routine practice. Third, this was a case-controlled study in which we could not control confounding factors that might affect the fracture healing. Thus, further studies, such as randomized controlled trial with a larger number of AFFs patients, are required to demonstrate the efficacy of this technique. However, to our knowledge, this study was the first study that used the DBM as an initial treatment in AFFs, and we believe that the local DBM augmentation would provide the better surgical outcomes and quality of life for the AFFs patients suffering from this fracture.

In conclusion, local DBM augmentation on the treatment of atypical subtrochanter femoral fracture is an interesting option. It is safe and effective in improving the fracture healing time and possibly reducing the fracture healing complication. Further researches are required to reveal its true efficacy and effectiveness in the treatment of AFFs.

## Figures and Tables

**Table 1:** Preoperative patients' characteristics.

	NDBM group (*n* = 9)	DBM group (*n* = 9)	*p* value
Age, year^+^	70 ± 7	63 ± 8	0.08
Female gender^■^	9 (100)	8 (89)	1.00
Fracture on right side^■^	4 (44)	6 (67)	0.64
BMI, kg/m^2^^+^	22.4 ± 2.4	24.1 ± 3.2	0.22
Comorbid diseases^■^			
Rheumatoid arthritis	0 (0)	2 (22)	0.47
Diabetes	2 (22)	1 (11)	1.00
Renal disease	1 (11)	2 (22)	1.00
Medications used^■^			
BPs	8 (89)	9 (100)	1.00
Statin	4 (44)	4 (44)	1.00
Steroids	1 (11)	3 (33)	0.58
PPIs	4 (44)	3 (33)	0.62
Duration of BPs before fracture, year^+^	8.9 ± 2.9^a^	7.6 ± 3.9	0.41
Preoperative laboratory values^+^			
Hb, g/dL	11.7 ± 1.4	12.0 ± 1.8	0.72
Albumin, g/L	37.5 ± 2.6	33.1 ± 7.4	0.14
CrCl, mL/minute/1.73 m^3^	68.4 ± 26.8	76.0 ± 31.8	0.59
Calcium, mg/dL	9.2 ± 0.7	8.6 ± 0.6	0.07
Inorganic phosphate, mg/dL	3.4 ± 0.3	3.3 ± 0.7	0.73
25-OH vitamin D, ng/mL	28.8 ± 9.3	27.8 ± 11.6	0.88
Total P1NP, ng/mL	46.4 ± 60.8	55.0 ± 64.8	0.8
*β*-cross laps, ng/mL	0.24 ± 0.21	0.23 ± 0.19	0.99
PTH, pg/mL	50.5 ± 47.9	51.2 ± 22.8	0.97
Follow-up time, week^+^	125 ± 60	72 ± 40	0.04^*∗*^

^+^Value presented as mean ± standard deviation. ^■^Value presented as number of patients (percentage).

BMI: body mass index; BPs: bisphosphonates; PPIs: proton pump inhibitors; Hb: hemoglobin.

CrCl: creatinine clearance; P1NP: procollagen type 1 N-terminal propeptide.

CTX: collagen type 1 C-telopeptide; PTH: parathyroid hormone.

^a^Calculated only from the patients receiving BPs. ^*∗*^Significant value as *p* < 0.05.

**Table 2:** Details of treatment and outcome on each patient.

Case number	Gender	Age (year)	Side	Comorbid diseases	DBM (mL)	Duration of BPs (year)	Indication of BPs	Anabolic treatment	Healing time (week)	Delayed union	Nonunion	Reoperation details
1	F	59	Rt	HLP	—	5	Osteopenia	STR	50.0	Yes	No	
2	F	78	Lt	HT, HLP	—	11	PMO	STR	39.4	Yes	No	
3	F	75	Rt	DM, HT, HLP	—	8	PMO	TPTD	112.3	Yes	Yes	ORIF with PF-LCP and IBG + NVFBG
4	F	67	Rt	HT, paroxysmal SVT	—	7	PMO	TPTD	43.1	Yes	No	
5	F	81	Lt	CKD, HT, HLP, ET	—	14	PMO	TPTD	88.1	Yes	No	
6	F	64	Lt	—	—	11	PMO	TPTD	13.7	No	No	
7	F	75	Rt	—	—	8	Osteopenia	STR	39.6	Yes	No	
8	F	66	Lt	Myasthenia gravis, HT	—	—	—	—	23.4	No	No	
9	F	65	Lt	HT, DLP, DM, asthma	—	7	PMO	TPTD	111.4	Yes	Yes	ORIF with ABP and DBM
10	M	59	Rt	Ventricular schwannoma	2	15	Osteoporosis	TPTD	52.3	Yes	No	
11	F	56	Rt	SLE, ESRD, DLP, HT, AVN	2	5	AVN	—	34.9	Yes	No	
12	F	66	Lt	ESRD s/p KT, HT, cardiomyopathy	1	1.5	PMO, GIOP	STR	13.9	No	No	
13	F	56	Rt	HT, OSA	1	6	PMO	TPTD	17.1	No	No	
14	F	64	Lt	DM, HT, RA, DLP	1	9	Osteopenia	TPTD	48.7	Yes	Yes	ORIF with ABP and DBM
15	F	64	Rt	DLP	2	10	PMO	TPTD	27.0	Yes	No	
16	F	79	Lt	HT, DLP	2	5	PMO	STR	19.4	No	No	
17	F	70	Rt	HT, DLP, peptic ulcer	1	7	PMO	TPTD	26.1	No	No	
18	F	57	Rt	—	1	10	PMO	TPTD	13.9	No	No	

HLP: hyperlipidemia; HT: hypertension; DM: diabetes; SVT: supraventricular tachycardia; CKD: chronic kidney disease.

ET: essential thrombocytosis; SLE: systemic lupus nephritis; ESRD: end-stage renal disease; AVNFH: avascular necrosis.

s/p KT: status postoperative kidney transplant; OSA: obstructive sleep apnea; RA: rheumatoid arthritis.

DBM: demineralized bone matrix; PMO: postmenopausal osteoporosis; GIOP: glucocorticoid induced osteoporosis.

STR: strontium ranelate; TPTD: teriparatide; ORIF: open reduction and internal fixation; PF-LCP: proximal femur locking compression plate.

ABP: angle blade plate; IBG: iliac bone graft; NVFBG: nonvascularized fibular bone graft.

**Table 3:** Postoperative outcomes.

	NDBM group (*n* = 9)	DBM group (*n* = 9)	*p* value
Postoperative alignment^+^			
Coronal plane^b^	−8.0 ± 7.1	−4.2 ± 3.8	0.18
Sagittal plane^c^	−5.2 ± 12.2	−6.3 ± 4.9	0.8
Neck-shaft angle^+^			
Fracture side	131 ± 8	132 ± 4	0.88
Normal side	136 ± 5	133 ± 5	0.25
Postoperative anabolic agent			
Teriparatide	5	6	1.00
Strontium ranelate	3	2	
Healing time, week^+^	57.9 ± 36.8	28.1 ± 14.4	0.04^*∗*^
Received teriparatide	73.7 ± 43.7	30.9 ± 16.1	0.09^w^
Not received teriparatide	38.1 ± 11.0	22.7 ± 10.9	0.13
Nonunion^■^	2 (2 : 0)	1 (1 : 0)	1.00
Delayed union^■^	7 (4 : 3)	4 (3 : 1)	0.33

^+^Value presented as mean ± standard deviation.

^■^Value presented as number of patients (received postoperative teriparatide: strontium ranelate).

^b^Negative and positive value meant varus and valgus angulation, respectively.

^c^Negative and positive value meant anterior and posterior angulation, respectively.

^*∗*^Significant value as *p* < 0.05.

^w^Calculated from *t*-test with a correction of unequal variance (Welch test).
